# Utilization of the internet for physical activity and diet information and its influence on pregnant women’s lifestyle habits in Qatar

**DOI:** 10.3389/fpubh.2023.1272109

**Published:** 2023-09-27

**Authors:** Ayman Al-Dahshan, Vahe Kehyayan, Noora Al-Kubaisi, Nagah Selim

**Affiliations:** ^1^Department of Medical Education, Hamad Medical Corporation, Doha, Qatar; ^2^University of Doha for Science and Technology, Doha, Qatar; ^3^Department of Clinical Affairs, Primary Health Care Corporation, Doha, Qatar; ^4^Department of Public Health and Preventive Medicine, Faculty of Medicine Cairo University, Cairo, Egypt

**Keywords:** pregnancy, lifestyle behavior, online information, health survey, middle east

## Abstract

**Background:**

Maintaining a healthy lifestyle during pregnancy is essential. With the increasing availability of the internet, pregnant women have access to a wealth of information related to physical activity (PA) and healthy diets.

**Objectives:**

The study aims to investigate the extent of internet usage by pregnant women attending antenatal clinics in Qatar to obtain information about PA and healthy diets, identify associated factors, and evaluate the impact of this information on their lifestyle habits.

**Methods:**

A cross-sectional study was conducted in six randomly selected public antenatal clinics in Qatar. Pregnant women aged ≥18 years in any trimester were recruited using a probability sampling technique. Data were collected through self-administered questionnaires. Descriptive and analytic statistics were employed for data analysis.

**Results:**

A total of 327 pregnant women (mean age of 29.5 years ± 4.0) completed and returned the questionnaire (response rate: 87.5%). Among the participants, 54.5% used the internet to obtain information about PA, while 72% sought online diet-related information. Factors such as age (26–35 years), tertiary education, employment status, and being primigravidae were associated with a higher likelihood of using the internet for healthy lifestyles information. Participants who reported using the internet for PA information a *great deal* were more likely to increase their PA during pregnancy compared to those who used it *somewhat or very little* (57.6, 26.8, and 5.0%, *p* < 0.001; respectively). Similarly, those who used the internet for diet information a *great deal* were more likely to increase their fruit and vegetable consumption compared to those who used it *somewhat, or very little* (84.2, 68.5, and 63.6%, *p* = 0.038; respectively). Also, participants who used the internet for diet information a *great deal* were more likely to decrease the amount of sugary and fatty foods they ate compared to women who used the internet *somewhat or very little* (61.4, 45.2, and 18.2%, *p* < 0.001; respectively) and (50.9, 30.4, and 18.2%, *p* = 0.001; respectively).

**Conclusion:**

Many pregnant women use the internet to access information about PA and diet, and greater use is associated with more positive lifestyle changes. This study provides useful information to consider when designing intervention programs for pregnant women about adopting and maintaining healthy lifestyles.

## Introduction

Pregnancy is considered a risk period for women as it is associated with poorer health behaviors including inadequate dietary habits and decreased physical activity (PA). As a result, pregnant women and their unborn child are at a greater risk of adverse health outcomes (1). Therefore, a woman’s health behaviors during pregnancy have lifelong implications for her own health and the health of her child (1).

The benefits of PA during pregnancy are numerous and include improvement of physical fitness, reduction in cesarean section rates, appropriate maternal and fetal weight gain, and management of gestational diabetes (2, 3). Also, PA during pregnancy is associated with improved psychological well-being and reduced risk of postpartum depressive symptoms (4). During pregnancy, if not contraindicated, a moderate-intensity aerobic exercise of 150 min throughout the week has been recommended (5). However, studies have shown that many pregnant women do not reach these recommendations, and that time allocated to PA tends to decrease during the course of pregnancy (6). Moreover, PA in pregnancy has been shown to be safe, desirable and of great benefit to most women. Hence pregnant women should be encouraged to continue or to initiate safe PA (5, 7).

In pregnancy, unhealthy diet and excessive gestational weight gain have been recognized as independent risk factors for maternal obesity and related pregnancy complications (1, 8). The offspring of these women are at higher risk of childhood overweight, obesity, and adult Type 2 Diabetes (9). Having a healthy diet during pregnancy helps women gain weight within international guidelines, potentially reducing the risk of related adverse outcomes (3, 10).

Studies have also indicated that online platforms have become popular resources for pregnant women to access information on PA and healthy diet (11–13). Current evidence suggests that online interventions can positively impact the dietary patterns of pregnant women. For instance, a systematic review conducted by Huang et al. (12) revealed that pregnant women who used online resources to manage their diet had a higher intake of fruits and vegetables than those who did not use online resources. In addition, a meta-analysis by Davenport et al. (14) established that online interventions may increase the time spent on moderate-intensity PA of pregnant women.

A recent study in Qatar reported that a majority of pregnant women (82%) were physically active; that is, they performed moderate PA more than twice a week for more than 20 min (15). As well, another study in Qatar evaluated the effect of an online pedometer program on PA participation among adults (16). The authors reported that such a program was effective in promoting PA, especially among women.

Overall, the internet has been shown to be a commonly used source of health information among pregnant women in Qatar (17). However, there is a lack of information about how pregnant women use the internet for information related to PA and healthy diets, and how this information could have an impact on their lifestyle. Therefore, we conducted this study with the following aims: (i) to explore how pregnant women in Qatar use the internet for information related to PA and healthy diets and their associated factors, and (ii) and to evaluate the impact of this information on women’s lifestyle habits.

## Methods

### Study design and setting

This study employed an analytical cross-sectional design and was conducted in public antenatal clinics within primary healthcare (PHC) centers in Qatar. At the time of the study, there were 20 public antenatal clinics available within PHC centers. These clinics were strategically distributed geographically to ensure comprehensive coverage for the diverse population with varying ethnic, cultural, social, and educational backgrounds. Additionally, these clinics served as the initial point of contact for patients seeking healthcare services within Qatar’s formal healthcare system (18). To achieve a representative sample, a random selection process was implemented, resulting in the inclusion of six antenatal clinics in the study. Data collection was carried out during the second half of 2019.

### Study population and sampling

The study population comprised expectant women, aged 18 years or older, in any trimester of pregnancy, who spoke English or Arabic, and presented at the antenatal clinic at one of the selected health centers during the study period. Participants were recruited through systematic random sampling, using the daily appointment list of the antenatal clinics until the required sample size of 324 individuals was reached.

### Sample size

The sample size was determined based on a 95% confidence level, 5% precision, and an estimated proportion of expectant women (70 ± 5%) accessing health-related information online. Using the formula for estimating sample size for proportions, a required sample size of approximately 324 participants was calculated.

### Data collection

Data were collected using an anonymized, self-administered questionnaire. Trained research assistants, who were registered nurses and midwives, were stationed at the chosen PHC centers to recruit and interview potential participants. They approached potential participants in the waiting areas of the antenatal clinics, provided information about the study, and invited them to participate voluntarily. Participants were assured that declining or withdrawing from the study would not affect the quality of care they received. Those who agreed to participate were signed a standardized informed consent form and were given the questionnaire to complete in either English or Arabic, according to their preference.

### Questionnaire

The questionnaire used in this study was developed by the researchers after conducting an extensive literature review. Experts in the field were consulted to evaluate the questionnaire’s clarity, relevance, and comprehensiveness. Additionally, face and content validity were assessed through a thorough expert review. Furthermore, test–retest reliability was evaluated, resulting in a coefficient of 0.9, which indicates a high level of reliability.

The questionnaire consisted of three sections. The first section included questions about socio-demographic characteristics such as age, education level, and occupation. The second section comprised questions about current pregnancy, including parity, gestational age, baby’s gender, and any health issues during the pregnancy. The third section contained questions about the frequency of internet use for PA and diet information, as well as changes in these behaviors resulting from online information. We defined PA as engaging in low-impact, moderate-intensity PA, such as walking, stationary cycling, prenatal yoga, and swimming, while excluding household duties. The questionnaire was translated and back-translated between English and Arabic by two independent translators proficient in both languages. It was piloted among 15 pregnant women to assess question clarity, interpretation, and completion time, and no modifications were required after the pilot phase. The pilot data were not included in the data analysis.

### Statistical analysis

Statistical analysis was performed using the Statistical Package for the Social Sciences (SPSS) version 23 (IBM Corp). Descriptive statistics were calculated for continuous and categorical variables where applicable. Pearson’s χ^2^-test and Fisher’s exact test were used to assess the association between the outcomes and the independent variables. The level of statistical significance was set at 0.05.

## Results

### Participant characteristics

Three hundred twenty-seven women completed and returned the survey questionnaire (response rate: 87.5%). Table 1 shows the background characteristics of the study participants. Their mean age was in the late twenties and a majority of the participants were multigravida and a little over half were in their second trimester. Most of the participants reported facing various pregnancy-related health issues including heartburn, morning sickness, low back pain, gestational diabetes, vomiting, anemia, urinary tract infection, and hypertension.

**Table 1:** Background characteristics of the study participants (*N* = 327).

Variable	*n* (%)
Age (year)	
18–25	48 (14.8)
26–35	246 (75.9)
36 or more	30 (9.3)
*Mean (SD)*	*29.5 (4.0)*
Level of education	
Up to secondary education	77 (23.6)
Tertiary education	249 (76.4)
Employment status	
Student	8 (2.4)
Housewife	189 (57.8)
Working	130 (39.8)
Parity	
Primigravida	88 (26.9)
Multigravida	239 (73.1)
Trimester	
First	18 (5.6)
Second	171 (52.8)
Third	135 (41.7)
Gender of fetus	
Male	121 (37.0)
Female	83 (25.4)
Unknown	123 (37.6)
Any health problems during current pregnancy^*^	
No	71 (21.7)
Yes	256 (78.3)

^*^included heartburn, morning sickness, low back pain, gestational diabetes, vomiting, anemia, urinary tract infection, and hypertension.

### Use of internet for physical activity and diet information

As shown in Table 2, a significant portion of participants reported using the internet to access information related to PA during their current pregnancy, ranging from “somewhat” to “a great deal.” A notable percentage stated that they increased their physical activity due to the internet-sourced information, while a smaller fraction decreased their physical activity. Almost half reported they did not change their PA during their pregnancy.

Regarding dietary information obtained from the internet during pregnancy, a substantial proportion of participants reported using online resources for this purpose. A majority increased their consumption of fruits and vegetables, reduced their intake of sugary foods, and decreased their consumption of fatty foods based on internet advice. Only a small percentage did not make any dietary changes during pregnancy (Table 2).

**Table 2:** Internet use for physical activity and diet-related information among study participants (*N* = 327).

Variable	*n* (%)
Extent of internet use for physical activity information	
A great deal	33 (10.3)
Somewhat	142 (44.2)
Very little	82 (25.5)
Not at all	64 (19.9)
Change of physical activity as a result of online information	
Increased	60 (19.0)
Decreased	26 (8.3)
Did not change	165 (52.4)
Not applicable (never used internet for PA information)	64 (20.3)
Extent of internet use for diet information	
A great deal	61 (19.0)
Somewhat	170 (53.0)
Very little	46 (14.3)
Not at all	44 (13.7)
Change in diet as a result of online information^*^	
Eat more fruits and vegetables	191 (61.0)
Eat less sugary food	119 (38.0)
Eat less fatty food	165 (52.4)
Did not change	22 (7.0)
Not applicable (never used internet for diet information)	44 (14.1)

^*^multiple responses.

### Factors associated with internet use for physical activity and diet information

In bivariate analysis, a statistically significant relationship was found between certain participants’ characteristics and the extent of internet use for PA and diet-related information (Table 3). For instance, women who were between the ages of 26 and 35, who were working, and who were primigravidae were significantly more likely to use the internet for PA information during pregnancy. Additionally, women who had tertiary education (*p* = 0.005), who were working (*p* = 0.004), and who were primigravidae (*p* = 0.009) were more likely to use the internet for diet-related information during pregnancy (Table 3).

**Table 3:** The relationship between participants’ characteristics and the use of internet for physical activity and diet-related information (*N* = 322).

Variable	*Extent of internet use for PA information*		*p* value	*Extent of internet use for diet information*		*p* value
	*Not at all to very little*	*Somewhat to a great deal*		*Not at all to very little*	*Somewhat to a great deal*	
	*n* (%)	*n* (%)		*n* (%)	*n* (%)	
Age (year)			0.049^*^			0.502
18–25	23 (50.0)	23 (50.0)		16 (34.8)	30 (65.2)	
26–35	103 (42.4)	140 (57.6)		64 (26.3)	179 (73.7)	
36 or more	19 (65.5)	10 (34.5)		8 (27.6)	21 (72.4)	
Nationality			0.568			0.270
Arab	79 (43.9)	101 (56.1)		46 (25.6)	134 (74.4)	
Non-Arab	65 (47.1)	73 (52.9)		43 (31.2)	95 (68.8)	
Level of education			0.072			0.005^*^
Up to secondary	41 (54.7)	34 (45.3)		31 (40.8)	45 (59.2)	
Tertiary	105 (42.9)	140 (57.1)		59 (24.2)	185 (75.8)	
Employment			0.003^*^			0.004^*^
Not working	100 (52.4)	91 (47.6)		65 (34.0)	126 (66.0)	
Working	46 (35.4)	84 (64.6)		25 (19.2)	105 (80.8)	
Gravida			0.008^*^			0.009^*^
Primigravida	29 (33.3)	58 (66.7)		15 (17.2)	72 (82.8)	
Multigravida	117 (50.0)	117 (50.0)		75 (32.1)	159 (67.9)	
Trimester			0.591			0.269
First	10 (55.6)	8 (44.4)		2 (11.1)	16 (88.9)	
Second	77 (46.4)	89 (53.6)		48 (28.9)	118 (71.1)	
Third	58 (43.3)	76 (56.7)		38 (28.4)	96 (71.6)	
Gender of fetus			0.504			0.532
Male	51 (43.2)	67 (56.8)		29 (24.4)	90 (75.6)	
Female	34 (42.5)	46 (57.5)		24 (30.0)	56 (70.0)	
I do not know	61 (49.6)	62 (50.4)		37 (30.3)	85 (69.7)	
Presence of health problems during this pregnancy			0.727			0.068
No	31 (43.7)	40 (56.3)		26 (36.6)	45 (63.4)	
Yes	115 (46.0)	135 (54.0)		64 (25.6)	186 (74.4)	

* Statistically significant result (*p* < 0.05).

### Changes in diet and physical activity as a result of online information

As shown in Table 4, the extent of internet use for PA-related information showed a significant positive association with women’s participation in PA. For instance, women who reported using the internet for PA information a great deal were more likely to increase their PA compared to women who used it somewhat, or very little (57.6, 26.8, and 5.0%, *p* < 0.001; respectively).

**Table 4:** Relationship between the extent of internet use for PA information and the resulting change in PA (*n* = 251).^a^

Extent of internet use for PA information	Total, *n* (%)	*Change in PA as a result of online information*			*p* value
		*Increased PA*	*Decreased PA*	*No change in PA*	
		*n* (%)	*n* (%)	*n* (%)	
A great deal	33 (100)	19 (57.6)	2^b^ (6.0)	12 (36.4)	<0.001*
Somewhat	138 (100)	37 (26.8)	14 (10.2)	87 (63.0)	< 0.001*
Very little	80 (100)	4 (5.0)	10 (12.5)	66 (82.5)	< 0.001*

^*^Statistically significant, ^a^participants who use the internet for physical activity information, ^b^Fisher’s exact test.

As shown in Table 5, the extent of internet use for diet-related information showed a significant positive association with women’s healthy eating behaviors. For instance, women who reported using the internet for diet information a great deal were more likely to increase their fruit and vegetable consumption during pregnancy compared to those who used it somewhat, or very little (84.2, 68.5, and 63.6%, *p* = 0.038; respectively). Also, women who used the internet for diet information a great deal were more likely to decrease the amount of sugary and fatty food they ate compared to those who used the internet somewhat or very little (61.4, 45.2, and 18.2%, *p* < 0.001; respectively) and (50.9, 30.4, and 18.2%, *p* = 0.001; respectively).

**Table 5:** Relationship between the extent of internet use for diet information and the resulting change in diet (*n* = 251).^a^

	*Changes in diet as a result of online information*									
	*Eat more fruits and vegetables*				*Eat less sugary food*				*Eat less fatty food*	
Extent of internet use for diet information	Total, *n* (%)	Yes, *n* (%)	No, *n* (%)	*p* value	Yes, *n* (%)	No, *n* (%)	*p* value	Yes, *n* (%)	No, *n* (%)	*p* value
A great deal	57 (100)	48 (84.2)	9 (15.8)	0.038*	35 (61.4)	22 (38.6)	<0.001*	29 (50.9)	28 (49.1)	0.001*
Somewhat	168 (100)	115 (68.5)	53 (31.5)		76 (45.2)	92 (54.8)		51 (30.4)	117 (69.6)	
Very little	44 (100)	28 (63.6)	16 (36.4)		8 (18.2)	36 (81.8)		8 (18.2)	36 (81.8)	

^*^Statistically significant, ^a^participants who use the internet for diet information.

## Discussion

This study investigated how pregnant women attending public antenatal clinics in Qatar utilize the internet to obtain information about PA and healthy diets and assessed the impact of this information on their lifestyle habits. The findings of this study indicate that the internet is being used to promote PA and healthy diets among pregnant women attending antenatal clinics in Qatar. Antenatal care facilities can play a crucial role in providing access to reliable, evidence-based lifestyle-related information (19). Around 54% of the participants in this study utilized the internet (*somewhat to a great deal*) for PA information, and of those, 20% reported an increase in their PA due to online information. These results align with a similar study conducted in the United States by Huberty et al. (20), which reported that approximately 44% of pregnant women used the internet for PA information to some extent, with 26% of them reporting an increase in PA as a result of this information.

In addition to PA information, our study found that a significant number of participants (72%) sought diet-related information online, leading to positive dietary changes such as increased fruit and vegetable consumption and reduced intake of sugary and fatty foods. These findings were consistent with a study in the United States (20), supporting the notion that online diet information can influence dietary behaviors in pregnant women. Current evidence suggests that online and mobile apps lifestyle-related interventions can positively impact the lifestyle of pregnant women (12, 14, 21–24). For instance, a 12-week study by Choi et al. (25) involving physically inactive pregnant women found that a mobile phone activity diary with self-monitoring led to an increase in PA compared to the control group. The authors concluded that motivated pregnant women may find online sources useful for assessing and promoting PA (25).

However, it should be noted that not all online sources led to lifestyle changes. In our study, almost half (52.4%) of the participants reported that they did not change their PA during their pregnancy as a result of online information. This finding aligns with the study by Huberty et al. (20) and the study Bagherzadeh et al. (21) which highlighted that motivation and trust in online information might be essential factors. Hence, healthcare professionals should emphasize the importance of PA and healthy eating and support pregnant women in their efforts to modify their lifestyles.

Regarding the factors associated with participants’ adoption of PA, age was identified as a factor influencing the use of the internet for PA information, with those between the ages of 26 and 35 being more likely to use the internet for such information. Other studies have shown inconsistent findings, possibly due to cultural differences in the countries where the studies were conducted (26–28). Our study also revealed that working (as a measure of income), level of education, and primigravidae were associated with seeking information about PA and diet. While some studies reported associations between exercise during pregnancy and income (29), the influence of education on internet use was inconsistent (26, 30). Moreover, our finding of an association between primigravidae and accessing online information to be more active was supported by Gaston and Cramp’s study (29).

Our study found that the extent of internet use for PA and dietary information was associated with more positive lifestyle changes. For example, women who reported using the internet for PA information to a great deal were significantly more likely to increase their PA compared to those who used it less often. Similarly, participants who reported using the internet for dietary information to a great deal were significantly more likely to increase their fruit and vegetable consumption while decreasing the intake of sugary and fatty foods compared to women who used the internet less frequently. These findings, although cross-sectional, provide valuable insights suggesting that the internet could be a valuable tool not only for increasing knowledge about PA and healthy diets but also for promoting these behaviors in pregnant women.

A qualitative study by Bagherzadeh et al. (21) demonstrated that accessing online information, combined with support from physicians, motivated pregnant women to adopt healthy lifestyles. Conversely, a recent qualitative study in Australia revealed that pregnant women perceived gaps in lifestyle-related knowledge provided by health professionals. Additionally, health professionals expressed difficulties in discussing sensitive topics, such as weight, with pregnant women and demonstrated limited familiarity with pregnancy-specific physical activity guidelines (31).

To sum up, the literature has provided rich information about the critical role of online information in pregnant women’s adoption of healthy lifestyles, including increasing PA and consuming healthy foods. However, there are reported variances in the factors associated with lifestyle changes, which could be attributed to differences in sampling methods, the validity and reliability of data collection tools, and cultural variations among the countries where the studies were conducted. Despite these variations, all studies consistently point to the increasing tendency of pregnant women to access online information with varying consequences.

### Strengths and limitations

This study boasts several strengths. Firstly, it was the pioneering research of its kind in Qatar and the region, examining pregnant women’s usage of the internet for PA and healthy diet information. It also evaluated associated factors and the impact of this information on women’s lifestyle habits. Moreover, the study achieved a commendable high response rate of 87.5%. Furthermore, the risk of sampling bias was minimized due to our use of a probability sampling technique when recruiting study participants. Finally, we believe our sample adequately represents pregnant women in Qatar, given the distribution of characteristics among the populations served by the six PHC centers.

However, we acknowledge several limitations as well. Firstly, our results may not be generalizable to all expectant women seeking antenatal services in Qatar’s private health sector, as our study focused on a sample attending publicly funded antenatal services. Future studies should compare internet use between these two populations to assess if they serve different groups of expectant women. Another limitation of this study is the absence of an assessment of participants’ daily physical activity (PA) levels. This gap underscores a potential avenue for exploration in future research. Lastly, our study recognizes potential response biases in self-reported data, including social desirability bias, recall bias, and cultural influences. While we implemented measures to minimize these biases, such as ensuring respondent anonymity and using clear language, some level of response bias may persist in the data.

### Implications for practice and research

The utilization of the internet to promote PA and healthy diets for pregnant women is gaining momentum. While challenges exist in using online platforms for educational purposes, they offer valuable opportunities to improve the health and well-being of pregnant women. Targeted interventions and reliable online resources tailored to their specific needs are essential. Positive associations between internet use frequency and behavior change, such as increased PA and improved dietary habits, demonstrate the potential of online platforms as effective tools for promoting healthy behaviors during pregnancy. Understanding participant characteristics helps tailor interventions for specific demographics, while further research is needed to evaluate the effectiveness of online interventions. Providing accurate, evidence-based resources and promoting health literacy will empower pregnant women to make informed decisions about their health and ensure healthier pregnancies overall.

## Conclusion

Our study reveals that a significant number of pregnant women use the internet to access information about PA and diet, and greater use is associated with more positive lifestyle changes. Women aged between 26 and 35, with tertiary education, employed, and primigravidae, were particularly inclined to use the internet for PA and diet-related information during pregnancy. Future research should focus on conducting longitudinal studies to assess the long-term impact of internet use on PA, dietary behaviors, and maternal and fetal outcomes, providing valuable insights into the sustainability and effectiveness of online interventions during pregnancy. Ultimately, recognizing the internet’s significance empowers pregnant women with accessible resources to make informed decisions and improve overall maternal and fetal well-being.

## Data availability statement

The original contributions presented in the study are included in the article/Supplementary material, further inquiries can be directed to the corresponding author.

## Ethics statement

The studies involving humans were approved by The ethical committee of the Primary Health Care Corporation. The studies were conducted in accordance with the local legislation and institutional requirements. The participants provided their written informed consent to participate in this study.

## Author contributions

AA-D: Conceptualization, Formal analysis, Methodology, Project administration, Writing – original draft. VK: Writing – review & editing. NA-K: Methodology, Writing – review & editing. NS: Supervision, Writing – review & editing.

## References

[1] Torloni MR Betran AP Horta BL Nakamura MU Atallah AN Moron AF. Prepregnancy BMI and the risk of gestational diabetes: a systematic review of the literature with meta-analysis. Obes Rev. (2009) 10:194–203. doi: 10.1111/j.1467-789X.2008.00541.x, PMID: 19055539

[2] Dipietro L Evenson KR Bloodgood B Sprow K Troiano RP Piercy KL. Benefits of physical activity during pregnancy and postpartum: An umbrella review. Med Sci Sports Exerc. (2019) 51:1292–302. doi: 10.1249/mss.0000000000001941 31095086 PMC6527310

[3] Hamann V Deruelle P Enaux C Deguen S Kihal-Talantikite W. Physical activity and gestational weight gain: A systematic review of observational studies. BMC Public Health. (2022) 22:1951. doi: 10.1186/s12889-022-14324-0, PMID: 36271388 PMC9585865

[4] Poyatos-Leon R Garcia-Hermoso A Sanabria-Martinez G Alvarez-Bueno C Cavero-Redondo I Martinez-Vizcaino V. Effects of exercise-based interventions on postpartum depression: a meta-analysis of randomized controlled trials. Birth. (2017) 44:200–8. doi: 10.1111/birt.12294, PMID: 28589648

[5] World Health Organization Physical activity (2022). Available at: https://www.who.int/news-room/fact-sheets/detail/physical-activity (Accessed May 4, 2023).

[6] Meander L Lindqvist M Mogren I Sandlund J West CE Domellöf M. Physical activity and sedentary time during pregnancy and associations with maternal and FETAL HEALTH OUTCOMES: an epidemiological study. BMC Pregnancy Childbirth. (2021) 21:166. doi: 10.1186/s12884-021-03627-6, PMID: 33639879 PMC7913456

[7] ACOG (2023). Physical activity and exercise during pregnancy and the postpartum period: ACOG committee opinion, number 804. Obstetrics and gynecology. U.S. National Library of Medicine. Available at: https://pubmed.ncbi.nlm.nih.gov/32217980/ (Accessed April 19, 2023). 10.1097/AOG.0000000000003772 32217980

[8] Langley-Evans SC Pearce J Ellis S. Overweight, obesity and excessive weight gain in pregnancy as risk factors for adverse pregnancy outcomes: a narrative review. J Hum Nutr Diet. (2022) 35:250–64. doi: 10.1111/jhn.12999, PMID: 35239212 PMC9311414

[9] Catalano PM Farrell K Thomas A Huston-Presley L Mencin P de Mouzon SH. Perinatal risk factors for childhood obesity and metabolic dysregulation. Am J Clin Nutr. (2009) 90:1303–13. doi: 10.3945/ajcn.2008.27416, PMID: 19759171 PMC2762159

[10] Marshall NE Abrams B Barbour LA Catalano P Christian P Friedman JE. The importance of nutrition in pregnancy and lactation: Lifelong consequences. Am J Obstet Gynecol. (2022) 226:607–32. doi: 10.1016/j.ajog.2021.12.035, PMID: 34968458 PMC9182711

[11] Zagaria AB Golubnitschaja O Costigliola V. Women's health and empowerment in the era of digital health: Opportunities and challenges for underserved women. Women's Health Issues. (2020) 30:346–9.

[12] Huang Y Gou L Chen X Chen T. Preliminary assessment of a mobile application to promote maternal and neonatal health: A quasi-experimental study in rural China. BMC Health Serv Res. (2020) 20

[13] Cannon S Lastella M Vincze L Vandelanotte C Hayman M. A review of pregnancy information on nutrition, physical activity and sleep websites. Women Birth. (2020) 33:35–40. doi: 10.1016/j.wombi.2018.12.007, PMID: 30905558

[14] Davenport MH Ruchat S-M Sobierajski F Poitras VJ Gray CE Yoo C. Impact of prenatal exercise on maternal harms, labour and delivery outcomes: A systematic review and meta-analysis. Br J Sports Med. (2018) 53:99–107. doi: 10.1136/bjsports-2018-099821, PMID: 30337349

[15] Naja S Elyamani R Al Ibrahim A Al Kubaisi N Itani R AbdulRouf P. The newest vital sign among pregnant women attending Women Wellness and Research Centre in Qatar: a cross-sectional study. BMC Pregnancy Childbirth. (2021) 21:73. doi: 10.1186/s12884-021-03542-w, PMID: 33478419 PMC7819321

[16] Al-Kuwari MG Al-Mohannadi AS Sayegh S. Effectiveness of "Step into health" program in Qatar: a pedometer-based longitudinal study. J Sports Med Phys Fitness. (2017) 57:1513–8. doi: 10.23736/S0022-4707.16.06716-5, PMID: 28004902

[17] Al-Dahshan A Chehab M Mohamed A Al-Kubaisi N Selim N. Pattern of internet use for pregnancy-related information and its predictors among women visiting primary healthcare in Qatar: A cross-sectional study. BMC Pregnancy Childbirth. (2021) 21:747. doi: 10.1186/s12884-021-04227-0, PMID: 34736404 PMC8568676

[18] Primary Health Care Corporation Primary Health Care Corporation: Health Centers. (2018). Available at: https://www.phcc.qa/portal_new/index/index.php?limit=home (Accessed February 19, 2022).

[19] Al-Dahshan A Chehab M Al-Kubaisi N Selim N. Reliability of online pregnancy-related information and associated feelings of worry among expectant women in Qatar. BMC Pregnancy Childbirth. (2022) 22:117. doi: 10.1186/s12884-022-04457-w, PMID: 35148714 PMC8840704

[20] Huberty J Dinkel D Beets MW Coleman J. Describing the use of the internet for health, physical activity, and nutrition information in pregnant women. Matern Child Health J. (2013) 17:1363–72. doi: 10.1007/s10995-012-1160-2, PMID: 23090284

[21] Bagherzadeh R Gharibi T Safavi B Mohammadi SZ Karami F Keshavarz S. Pregnancy; an opportunity to return to a healthy lifestyle: a qualitative study. BMC Pregnancy Childbirth. (2021) 21:751. doi: 10.1186/s12884-021-04213-6, PMID: 34740317 PMC8569967

[22] van Dijk MR Koster MP Oostingh EC Willemsen SP Steegers EA Steegers-Theunissen RP. A mobile app lifestyle intervention to improve healthy nutrition in women before and during early pregnancy: Single-center Randomized Controlled Trial. J Med Internet Res. (2020) 22. doi: 10.2196/15773 PMC7260659 32412417

[23] Ghahramani A de Courten M Prokofieva M. The potential of social media in health promotion beyond creating awareness: an integrative review. BMC Public Health. (2022) 22:1–13. doi: 10.1186/s12889-022-14885-0 36544121 PMC9770563

[24] Kiani N Pirzadeh A. Mobile-application intervention on physical activity of pregnant women in Iran during the COVID-19 epidemic in 2020. J Educ Health Promot. (2021) 10:328. doi: 10.4103/jehp.jehp_56_21 34761014 PMC8552263

[25] Choi J Lee J Vittinghoff E Fukuoka Y. MHealth physical activity intervention: A randomized pilot study in physically inactive pregnant women. Matern Child Health J. (2015) 20:1091–101. doi: 10.1007/s10995-015-1895-7 PMC4826820 26649879

[26] Rezaee R Ravangard R Amani F Dehghani Tafti A Shokrpour N Bahrami MA. Healthy lifestyle during pregnancy: uncovering the role of online health information seeking experience. PLoS One. (2022) 17:e0271989. doi: 10.1371/journal.pone.0271989, PMID: 35913949 PMC9342740

[27] Scaioli G Bert F Galis V Brusaferro S De Vito E La Torre G. Pregnancy and internet: Sociodemographic and geographic differences in e-health practice. results from an Italian multicenter study. Public Health. (2015) 129:1258–66. doi: 10.1016/j.puhe.2015.06.012, PMID: 26210071

[28] Nawabi F Krebs F Lorenz L Shukri A Alayli A Stock S. Understanding determinants of pregnant women’s knowledge of lifestyle-related risk factors: a cross-sectional study. Int J Environ Res Public Health. (2022) 19:658. doi: 10.3390/ijerph19020658, PMID: 35055480 PMC8775702

[29] Gaston A Cramp A. Exercise during pregnancy: a review of patterns and determinants. J Sci Med Sport. (2011) 14:299–305. doi: 10.1016/j.jsams.2011.02.006 21420359

[30] Sayakhot P Carolan-Olah M. Internet use by pregnant women seeking pregnancy-related information: a systematic review. BMC Pregnancy Childbirth. (2016) 16:65. doi: 10.1186/s12884-016-0856-5, PMID: 27021727 PMC4810511

[31] Knight-Agarwal C Minehan M Cockburn B Cashel S Takito MY. Different experiences of weight management and physical activity during pregnancy—a qualitative study of women and healthcare professionals in Australia. Int J Qual Stud Health Well Being. (2023) 18. doi: 10.1080/17482631.2023.2202973, PMID: 37099751 PMC10134913

