# Supplementary material for: Utilization of the internet for physical activity and diet information and its influence on pregnant women’s lifestyle habits in Qatar
Source: Front Public Health. 2023 Sep 27;11:1272109. doi: 10.3389/fpubh.2023.1272109 (PMC10564989; doi:10.3389/fpubh.2023.1272109)
Supplement: Supplementary file 1 [file Data_Sheet_1.PDF]

Participant Number	Date of data collection	Health Centre Name
	/ /	

Using the Internet for Diet and Physical Activity Information during pregnancy

استخدام الإنترنت للحصول على معلومات حول النظام الغذائي والنشاط البدني أثناء الحمل

1	**What is your age?** (in whole year)<br>**كم عمرك؟** (بالسنين الكاملة)			
2	**What is your nationality?**<br>**ماهي جنسيتك؟**			
3	**What is your educational level?**	❑ Read and write<br>❑ Primary<br>❑ Secondary<br>❑ College/University	استطيع القرآءة والكتابة ❑<br>إبتدائي ❑<br>ثانوي ❑<br>كلية / جامعي ❑	**ما هو مؤهلك العلمي؟**
4	**What is your current occupational status?**	❑ Student<br>❑ Employed<br>❑ Housewife	طالبة ❑<br>موظفة ❑<br>ربة منزل ❑	**ما هو وضعك الوظيفي الحالي؟**
5	**How many living children do you have?**	❑ No children<br>❑ One child<br>❑ Two children<br>❑ Three children<br>❑ Four children<br>❑ Five children<br>❑ Six children or more	لا يوجد أطفال ❑<br>طفل واحد ❑<br>طفلان إثنان ❑<br>ثلاثة أطفال ❑<br>أربعة أطفال ❑<br>خمسة أطفال ❑<br>ستة أطفال أو أكثر ❑	**كم عدد الأطفال لديك؟**
6	**What is the number of your current pregnancy** (including previous abortion(s) if any)?	❑ The first<br>❑ The second<br>❑ The third<br>❑ The fourth<br>❑ The fifth<br>❑ More than that,<br>(please specify)	الأول ❑<br>الثاني ❑<br>الثالث ❑<br>الرابع ❑<br>الخامس ❑<br>أكثرمن ذلك, ❑<br>(الرجاء التحديد)........................	**ما هو ترتيب الحمل الحالي** (بما في ذلك الإجهاضات السابقة إذا حدثت)؟
8	**In which week of pregnancy are you currently?**	❑ Week number	الأسبوع رقم .... ❑	**في أي اسبوع من الحمل أنت حاليا؟**
9	**What is the gender of the baby?**	❑ Male<br>❑ Female<br>❑ I don't know	ذكر ❑<br>أنثى ❑<br>لا أعلم ❑	**ما هو جنس الجنين ؟**

10	**What health problems do you have during your current pregnancy?**<br>**(Please select all applicable)**	❑ Morning sickness<br>❑ Vomiting<br>❑ Heartburn<br>❑ Anaemia<br>❑ Gestational diabetes<br>❑ High/low blood pressure<br>❑ Urinary tract infection<br>❑ Vaginal bleeding<br>❑ Low back pain<br>❑ Depression or Anxiety<br>❑ Preeclampsia<br>❑ Other (please specify)<br>……………………………….<br>❑ **I have no health problem**	❑ الغثيان الصباحي<br>❑ التقيء<br>❑ حرقة المعدة<br>❑ فقر لدم<br>❑ سكري الحمل<br>❑ ارتفاع أوانخفاض ضغط الدم<br>❑ التهاب المسالك البولية<br>❑ النزيف المهبلي<br>❑ آلام أسفل الظهر<br>❑ الإكتئاب أو القلق<br>❑ تسمم الحمل<br>❑ مشاكل اخرى (الرجاء التحديد)<br>……………………………….<br>❑ **لا أعاني من أي مشاكل صحية**	**ما هي المشاكل الصحية التي تعانين منها خلال فترة الحمل الحالي؟**<br>**(الرجاء اختيار كل ما ينطبق)**
11	**How often did you exercise regularly (≥ 20 min) <u>before</u> pregnancy?**<br>(besides house duties)	❑ Everyday<br>❑ 3-4 times a week<br>❑ 1-2 times a month<br>❑ I don't exercise	❑ يوميا<br>❑ ٣-٤ مرات في الأسبوع<br>❑ مرة او مرتين في الشهر<br>❑ لا أمارس الرياضة	**كم عدد المرات التي تمارسين فيها نشاط رياضي منتظم (≤ ٢٠ دقيقة) <u>قبل</u> فترة الحمل ؟**<br>(باستثناء الأعمال المنزلية)
12	**Since becoming pregnant, has your physical activity?**<br>physical activity means doing things like walking, pedaling on a stationary bike, practicing prenatal yoga, or swimming. Remember, we're not counting regular household chores in this.	❑ Increased<br>❑ Decreased<br>❑ Didn't change	❑ ازداد<br>❑ قل<br>❑ لم يتغير	**منذ بداية الحمل، كيف تغير نشاطك البدني؟**<br>النشاط البدني يعني القيام بأشياء مثل المشي، ركوب الدراجة الثابتة، ممارسة اليوغا الحمل، أو السباحة. تذكري، لا نأخذ في اعتبارنا أعمال المنزل اليومية في هذا.
13	**To what extent do you use the internet for information on physical activity/exercise during pregnancy?**	❑ Never<br>❑ Rarely<br>❑ Sometimes<br>❑ Frequently	❑ نهائياً<br>❑ نادرا<br>❑ بعض الأحيان<br>❑ بشكل متكرر	**إلى أي مدى تستخدمين الإنترنت للحصول على معلومات حول النشاط البدني / التمارين الرياضية أثناء الحمل؟**
14	**As a result of the information that you found online, has your physical activity؟**	❑ Increased<br>❑ Decreased<br>❑ Didn't change<br>❑ Not applicable	❑ ازداد<br>❑ قل<br>❑ لم يتغير<br>❑ لا ينطبق	**نتيجة للمعلومات التي عثرت عليها عبر الإنترنت ، كيف تغير نشاطك البدني أثناء الحمل ؟**

15	**To what extent did you use the internet for information on foods you eat during pregnancy?**	❑ Never<br>❑ Rarely<br>❑ Sometimes<br>❑ Frequently	❑ نهائياً<br>❑ نادرا<br>❑ بعض الأحيان<br>❑ بشكل متكرر	**إلى أي مدى قمتي باستخدام الإنترنت للحصول على معلومات حول الأطعمة التي تتناوليها أثناء الحمل؟**
	**If you answered "NEVER", kindly skip question 15.1**		**إذا كانت إجابتك " نهائيا "، يرجى تخطي السؤال رقم 15.1**	
15.1	**As a result, if you changed the foods you consume during pregnancy (choose all applicable)**	❑ Eat more fruit and vegetable<br>❑ Eat less high-sugar food<br>❑ Eat less fatty food<br>❑ No changes made<br>❑ Others……………………….	❑ تناول المزيد من الفاكهه والخضروات<br>❑ تناول كميات أقل من الطعام الدهني<br>❑ تناول كميات أقل من الطعام عالي السكر<br>❑ لم يتم إجراء أي تغييرات<br>❑ فعل أخر………………………………	**نتيجة لذلك ، إذا قمتي بتغيير الأطعمة التي تتناوليها أثناء الحمل**<br>**(الرجاء اختيار كل ما ينطبق)**

Thank you for your valuable participation **شكراً جزيلا على مشاركتكم القيّمة**
